# Optimization of Fermentation Conditions and Rheological Properties of Exopolysaccharide Produced by Deep-Sea Bacterium *Zunongwangia profunda* SM-A87

**DOI:** 10.1371/journal.pone.0026825

**Published:** 2011-11-11

**Authors:** Sheng-Bo Liu, Li-Ping Qiao, Hai-Lun He, Qian Zhang, Xiu-Lan Chen, Wei-Zhi Zhou, Bai-Cheng Zhou, Yu-Zhong Zhang

**Affiliations:** State Key Laboratory of Microbial Technology, Marine Biotechnology Research Center, Shandong University, Jinan, People's Republic of China; University of Kansas, United States of America

## Abstract

*Zunongwangia profunda* SM-A87 isolated from deep-sea sediment can secrete large quantity of exopolysaccharide (EPS). Response surface methodology was applied to optimize the culture conditions for EPS production. Single-factor experiment showed that lactose was the best carbon source. Based on the Plackett–Burman design, lactose, peptone and temperature were selected as significant variables, which were further optimized by the steepest ascent (descent) method and central composite design. The optimal culture conditions for EPS production and broth viscosity were determined as 32.21 g/L lactose, 8.87 g/L peptone and an incubation temperature of 9.8°C. Under these conditions, the maximum EPS yield and broth viscosity were 8.90 g/L and 6551 mPa•s, respectively, which is the first report of such high yield of EPS from a marine bacterium. The aqueous solution of the EPS displayed high viscosity, interesting shearing thinning property and great tolerance to high temperature, a wide range of pH, and high salinity.

## Introduction

Many microorganisms can synthesize exopolysaccharides (EPSs) and excrete them out of cell either as soluble or insoluble polymers. These EPSs not only can protect the microorganisms themselves, but also can be applied in many biotechnological applications, such as textile, pharmaceutical, cosmetics, food, metal mining, oil recovery and metal recovery [1]. Common microbial EPSs, such as xanthan and dextran, have been used as commercial products for many years. Because of their novel properties, the bioactive microbial polysaccharides β-D-glucans and bacterial cellulose have been used for immune modulation and tumouristasis and audio membranes [2]. Thus, microbial EPSs have attracted more attentions from scientific and industrial communities.

The marine environment, which represents 70% of the earth's surface and 90% of the volume of its crust, offers vast source of natural products [1]. Most microbial cells in the marine environment are surrounded by EPSs, which may assist microbial communities to endure extremes of temperature, salinity, and nutrient availability [3]. Because of the attractive chemical and rheological properties of the EPSs produced by marine microorganisms, studies have been conducted to investigate their potential applications in biotechnology, environmental protection, food industry, and so on [3], [4].

The yield and quality of microbial EPSs are greatly affected by the nutritional and environmental conditions [5]. For instance, the EPS yield of strain *Rhizobium tropici* was significantly influenced by carbon source, carbon : nitrogen ratio, and pH [6]. The molecular weight of the alginate from *Pseudornonas fluorescens* produced on fructose was much higher than that produced on glucose [7]. Response surface methodology (RSM) is an efficient statistically strategy for designing experiments, building models, searching optimum conditions of factors for desirable responses and evaluating the relative significance of several affecting factors even in the presence of complex interactions [8], [9]. Plackett-Burman (PB) design [10] is usually used as the first step to screen main factors from a number of process variables, which is generally followed by the steepest ascent (descent) method and central composite design (CCD) [11].

*Zunongwangia profunda* SM-A87 was isolated from the deep-sea sediment of the southern Okinawa Trough area at a water depth of 1245 m with *in situ* temperature of 4.7°C [12]. Previous study showed that SM-A87 contains two gene clusters for polysaccharide synthesis and export and can produce large quantity of EPS [13]. As an adsorbent, the EPS from strain SM-A87 has optimum biosorption capacities for Cu(II) and Cd(II) [14]. Moreover, its aqueous solution has good rheological properties for enhanced oil recovery [15]. Because of the biotechnological potential of the EPS from SM-A87, it is necessary to improve the EPS yield of strain SM-A87. In this study, RSM was used to optimize the fermentation conditions for EPS production by strain SM-A87. In addition, the rheological properties of the crude EPS solution were studied for exploring its potential applications in biotechnology.

## Materials and Methods

### Strain and media

The deep-sea sediment sample was retrieved from near the southern Okinawa Trough at a water depth of 1245 m during the IMAGES XII, MD-147-Marco Polo Leg 2 cruise of the R/V Marion Dufresne of the French Polar Institute (IPEV). The strain SM-A87 (CCTCC AB 206139^T^ = DSM 18752) isolated from the deep-sea sediment sample, is a species of *Bacteroidetes* and was proposed to represent a new genus of *Flavobacteriaceae* [12]. It was renamed *Zunongwangia profunda* in the *International Journal of Systematic and Evolutionary Microbiology* (IJSEM) Validation List no. 116 [11]. The isolated strain was cultured at 30°C for 3 d on an marine agar (MA) medium composed of 10 g/L peptone, 5 g/L yeast extract (both Oxoid, UK), 15 g/L agar and artificial sea water, and then stored at 4°C, which was sub-cultured every 2 months. The basal medium for EPS production contained (g/L): glucose 30, peptone 10, yeast extract 5 and artificial sea water; the initial pH of the medium was adjusted to 7.5. All chemicals used in this study were of analytical reagent grade.

### Inoculum preparation and flask fermentation

For inoculum preparation, strain SM-A87 was inoculated into an Erlenmeyer flask (250 mL) containing 50 mL basal medium and incubated for 60 h at 15°C, 200 rpm to logarithmic phase. For fermentation, a 2% (v/v) inoculum was added to the flask (500 mL) containing 100 mL basal medium, and then the flask was incubated at 15°C, 200 rpm for 6 d.

### Determination of EPS yield and broth viscosity

Samples taken from growing cultures were mixed with two volumes of cold absolute ethanol, and centrifuged for 10 min at 10,000 rpm. To remove impurities of low molecular weight, the precipitation was washed twice using absolute ethanol and followed by centrifugation. The precipitation was dissolved in deionized water and the EPS concentration of the solution was determined using the phenol-sulfuric acid method [16]. All experimental data given below were based on mean values obtained from three parallel samples, and the same procedure was done with the uninoculated medium as control.

The viscosity of the samples collected from the fermentation flasks was performed on a Brookfield viscometer (model LVDV-II+P; Brookfield Engineering Laboratories, USA) at 25°C. All assays were carried out with the small sample adapter and the spindle S16.

### Effect of different carbon sources on EPS production

Effect of carbon sources on EPS production of strain SM-A87 was studied by adding 30 g/L different sugars, namely glucose, mannose, lactose, maltose, and sucrose, in the basic marine medium [17].

### Screening of significant variables using PB design

The PB experimental design was applied to screen the significant variables that influence EPS production and broth viscosity. Eight variables of medium composition and culture conditions were tested at low (−1) and high (+1) levels based on PB matrix design, which is a fraction of a two-level factorial design and allows the investigation of *n−1* variables in at least *n* experiments [10]. In order to estimate the experimental error and check the adequacy of the first-order model, three insignificant dummy variables were added to the variables of real interest. The lower and higher levels of each variable and the design matrix are shown in Table 1. PB experimental design is based on the first-order model:
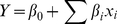
(1)where *Y* is the predicted response, *β_0_* is the model intercept, *β_i_* is the linear coefficient and *x_i_* is the level of the independent variable. Both EPS production and broth viscosity were measured in triplicate and the average value was taken as the response. The variables significant at 95% level (*P*<0.05) were considered to have significant effect on EPS production or broth viscosity and thus used for further optimization.

**Table 1:** Plackett–Burman experimental design for screening of culture conditions affecting EPS production and broth viscosity of strain SM-A87^a^.

Run	*X*_1_	*X*_2_	*X*_3_	*X*_4_	*X*_5_	*X*_6_	*X*_7_	*X*_8_	*X*_9_	*X*_10_	*X*_11_	EPS production (g/L)	Broth viscosity (mPa•s)
1	30	15	5	3	8.5	25	2	6	−1	1	−1	4.35	519.9
2	20	15	7.5	2	8.5	25	3	6	−1	−1	1	2.65	116
3	30	10	7.5	3	7.5	25	3	8	−1	−1	−1	5.77	2148
4	20	15	5	3	8.5	15	3	8	1	−1	−1	4.09	863.8
5	20	10	7.5	2	8.5	25	2	8	1	1	−1	3.04	299.9
6	20	10	5	3	7.5	25	3	6	1	1	1	4.07	451.9
7	30	10	5	2	8.5	15	3	8	−1	1	1	7.01	3521
8	30	15	5	2	7.5	25	2	8	1	−1	1	3.69	671.9
9	30	15	7.5	2	7.5	15	3	6	1	1	−1	5.75	1564
10	20	15	7.5	3	7.5	15	2	8	−1	1	1	4.08	463.9
11	30	10	7.5	3	8.5	15	2	6	1	−1	1	6.97	2323.667
12	20	10	5	2	7.5	15	2	6	−1	−1	−1	4.64	939.8

a *X*_1_, lactose (g/L); *X*_2_, peptone (g/L); *X*_3_ , yeast extract (g/L); *X*_4_, sea salt (%); *X*_5_, pH; *X*_6_, temperature (°C) ; *X*_7_, inoculum size (%); *X*_8_ , time (day); *X*_9_, *X*_10_ and *X*_11_, dummy variables.

### Path of steepest ascent (or descent)

Because initial estimates of operating conditions for the system are usually far from the actual optimum, a method is needed to move rapidly to the general vicinity of the optimum via experimentation. The method of steepest ascent (descent) is a procedure for moving sequentially along the path of steepest ascent (descent), that is, along the path of the maximum increase (decrease) in the response [18]. For a first-order model in PB design, the contours of the response surface are a series of parallel lines. The direction of steepest ascent is the one in which the response *Y* increases most rapidly, and this direction is normal (perpendicular) to the fitted response surface contours. The path of steepest ascent (descent) is usually the line through the center of the region of interest and normal to the fitted surface contours [19]. Thus, the steps along the path are proportional to the regression coefficients *β_i_*. As shown in Table 2, the path of steepest ascent (descent) started from the center of the chosen variables in the PB design, and moved along the path in which *X*_1_, *X*_2_, *X*_6_ were moved by 6, −1.94, −5, respectively.

**Table 2:** Experiment design of steepest ascent and corresponding response^a^.

	Lactose (g/L)	Peptone (g/L)	Temperature (°C)	EPS production (g/L)	Broth viscosity (mPa•s)
Base point^b^	25.00	12.50	20.00		
Origin step unit^c^	5.00	2.50	5.00		
Slope^d^	0.897	−0.579	−0.748		
Proportion^e^	4.49	−1.45	−3.74		
New unit^f^	6.00	−1.94	−5.00		
Experiment 1	25.00	12.50	20.00	4.84	2203.67
Experiment 2	31.00	10.56	15.00	3.17	144
Experiment 3	37.00	8.62	10.00	7.35	4771
Experiment 4	42.00	6.68	5.00	1.29	24

a *X*_1_, lactose; *X*_2_, Peptone; *X*_6_ , temperature.

b zero level in the PB design in Table 1.

c range of the unity level.

d estimated coefficient ratio from Eq. (1).

e origin step unit × slope.

f Proportion ×1.337, where 1.337 is a factor determined by experimenter based on process knowledge or other practical consideration, and 1.337 is appropriate in this example.

### Optimization of significant variables using CCD

To find the optimal cultivation conditions for EPS production, CCD with five coded levels was used for locating the true optimum conditions of lactose, peptone and temperature. For the three factors, this trial was essentially a full 2^3^ factorial design with six axial points (α = 1.68) and six replication of the center points, resulting in a total number of 20 experiments. The levels of the variables and the experimental design are shown in Table 3. The results of CCD were expressed as the following second-order polynomial Eq. 2 using a multiple regression technique.
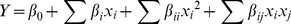
(2)where *Y* is the predicted response, *β*_0_ the intercept term, *βi* the linear coefficients, *β_ii_* the quadratic coefficients, *β_ij_* the interactive coefficients, and *x_i_* and *x_j_* the coded independent variables [8].

**Table 3:** The matrix of the CCD experiment and the corresponding experimental data.

Run	*X*_1_ (lactose)		*X*_2_ (peptone)		*X*_3_ (temperature)		EPS production (g/L)	Broth viscosity (mPa•s)
	Coded level	Real level (g/L)	Coded level	Real level (g/L)	Coded level	Real level (°C)		
1	−1	30	−1	6.5	−1	8	6.81	3947
2	+1	40	−1	6.5	−1	8	6.78	3695
3	−1	30	+1	10.5	−1	8	8.25	5495
4	+1	40	+1	10.5	−1	8	7.04	4515
5	−1	30	−1	6.5	+1	12	7.05	4551
6	+1	40	−1	6.5	+1	12	7.58	4747
7	−1	30	+1	10.5	+1	12	6.97	4301
8	+1	40	+1	10.5	+1	12	6.58	3377.5
9	−1.68	26.6	0	8.5	0	10	8.41	6503
10	+1.68	43.4	0	8.5	0	10	7.71	5079
11	0	35	−1.68	5.14	0	10	7.25	4579
12	0	35	+1.68	11.86	0	10	7.13	4559
13	0	35	0	8.5	−1.68	6.64	6.81	3923
14	0	35	0	8.5	+1.68	13.36	6.93	4091
15	0	35	0	8.5	0	10	8.39	6663
16	0	35	0	8.5	0	10	8.70	6239
17	0	35	0	8.5	0	10	8.60	6799
18	0	35	0	8.5	0	10	8.62	6443
19	0	35	0	8.5	0	10	8.47	6415
20	0	35	0	8.5	0	10	8.49	6611

### Statistical analysis

Experimental designs and the polynomial coefficients were calculated and analyzed using a trial version of Design-Expert software (version 8.0.4, Stat-Ease Inc., Minneapolis, USA). Statistical analysis of the model was performed to evaluate the analysis of variance (ANOVA).

### Preparation and properties analysis of crude EPS solution

Because the broth from flask fermentation was too thick to remove the bacterial cells by centrifugation, two volumes of cold absolute ethanol were directly added into the broth after fermentation. The mixture was kept overnight at 4°C, and then centrifuged for 10 min at 10,000 rpm. The precipitate was washed twice using cold absolute ethanol and dried in a desiccator at 60°C for 24 h. In order to remove proteins, the dried precipitation was dissolved in deionized water (1%, w/v), and was incubated for 6 h at 50°C, 200 rpm on a rotary shaker after 5 U/mL alkaline protease (Novozymes, Denmark) was added [20]. After re-precipitation using cold absolute ethanol and drying in a desiccator, the crude EPS was collected. To prepare the crude EPS solution of different concentration (0.2%, 0.4%, 0.6%, 0.8%, 1.0%, and 1.2% (w/v)), the crude EPS was dissolved into hot deionized water (80°C) under 1 h strong stirring of 400 rpm and further stirred for 12 h at room temperature. The viscosity of the crude EPS solution under different shear rates (1, 2, 10, 15, 20, 40, 60 rpm) was measured using a Brookfield viscometer with the small sample adapter and the spindle S16 at 25°C. To investigate the stability of the EPS from strain SM-A87 at different temperatures, the crude EPS solution was incubated for 10 min at 10, 20, 30, 40, 50, 60, 70 and 80°C, respectively. HCl (10 M) and NaOH (10 M) were used to adjust the pH values of the EPS solutions while 200 g/L NaCl and 200 g/L CaCl_2_ were used to adjust the different electrolyte concentrations. The viscosity of the above EPS solution was measured using a Brookfield viscometer with the small sample adapter and the spindle S16 at a shear rate of 20 rpm.

## Results

### Effect of different carbon sources on EPS production

**Figure 1:**
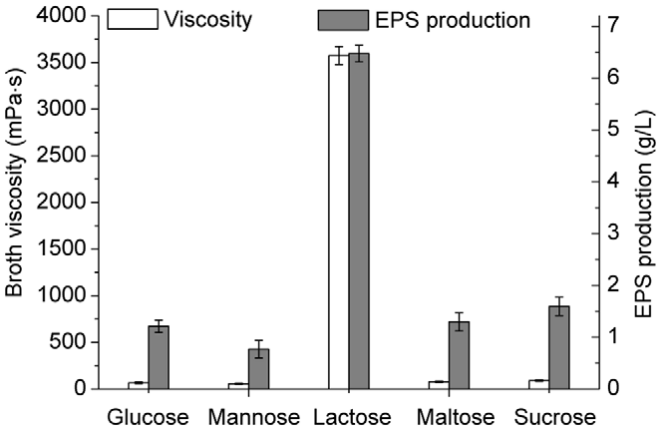
Effect of carbon sources on the EPS production and broth viscosity of strain SM-A87. The concentration of each carbon source was 30 g/L. The graph shows data from triplicate experiments (mean ± S.D.).

The effect of five different carbon sources on the EPS production and broth viscosity is summarized in Figure 1. The maximum EPS production (about 6.47 g/L) was detected when lactose was served as carbon source, which had significant advantage over the other four carbon sources.

### PB design

In order to find out the key factors significantly affecting the EPS production of strain SM-A87, the relative significance of eight variables, lactose, peptone, yeast extract, sea salt, initial pH, inoculum size, and incubation time were investigated using PB design. The ANOVA of PB design for EPS production and broth viscosity is shown in Table 4. The determinant of coefficient *R* ^2^ of the first-order model were 0.9890 for EPS production and 0.9723 for broth viscosity, indicating that the data variability could be explained by the models very well. The *p*-values of the models were 0.0075 and 0.0288, indicating that the models were significant. Usually, a model term is considered to be significant when its value of “*p*-value” is less than 0.05 [9]. In this case, lactose, peptone and temperature were significant model terms, indicating that these three variables were the greatest important variables for EPS production and broth viscosity. Thus, lactose, peptone and temperature were selected for further optimization using path of steepest ascent (or descent) and CCD. By applying multiple regression analysis on the experimental data, the following first-order polynomial equation was established to explain the EPS production and broth viscosity:
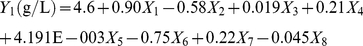
(3)

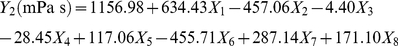
(4)where *Y*_1_ was the EPS production, *Y*_2_ the broth viscosity, *X*_1_ the lactose, *X*_2_ the peptone, *X*_3_ the yeast extract, *X*_4_ the sea salt, *X*_5_ the pH, *X*_6_ the temperature, *X*_7_ the inoculum size, and *X*_8_ the time.

**Table 4:** Identification of significant variables for EPS production and broth viscosity of strain SM-A87 using PB design.

Variables	EPS production^a^				Broth viscosity^b^			
	Coefficient estimate	% Contribution	*F* value	*p*-value Prob > *F*	Coefficient estimate	% Contribution	*F* value	*p*-value Prob > *F*
Model	−	−	33.62	0.0075^c^	−	−	13.15	0.0288^c^
Intercept	4.60	−	−	−	1156.98	−	−	−
Lactose	0.90	43.31	120.52	0.0016^c^	634.43	41.40	44.80	0.0068^c^
Peptone	−0.58	18.49	50.28	0.0058^c^	−457.06	21.49	23.25	0.0170^c^
Yeast extract	0.019	0.019	0.052	0.8342	−4.40	1.994E-003	2.157E-003	0.9659
Sea salt	0.21	2.50	6.80	0.0799	−28.45	0.083	0.090	0.7836
pH	4.191E-003	9.676E-004	2.632E-003	0.9623	117.06	1.41	1.53	0.3047
Temperature	−0.75	30.86	83.92	0.0028^c^	−455.71	21.36	23.11	0.0171^c^
Inoculation	0.22	2.61	7.10	0.0761	287.14	8.48	9.18	0.0563
Time	−0.045	0.11	0.31	0.6184	171.10	3.01	3.26	0.1688

a *R* ^2^ = 0.9890.

b *R* ^2^ = 0.9723.

c Model terms are significant.

### Path of steepest ascent (or descent)

Based on the aforementioned first-order model equation (Eq. 3) and the obtained three important effect variables above, the path of steepest ascent (descent) was determined to find proper direction of changing the variables according to the sign of the main effects to improve EPS production and broth viscosity. The path of steepest ascent started from the center of the PB design and moved along the path in which lactose concentration increased, while peptone concentration and temperature decreased. Table 2 shows the design and experimental results. It was evident that the highest response was reached at the third step when lactose concentration was 37.00 g/L, peptone concentration was 8.62 g/L, and incubation temperature was 10.00°C, suggesting that this point was near the region of maximum production response.

### CCD and response surface analysis

The optimal level of the key variables (lactose, peptone and temperature) and the effect of their interactions on EPS production and broth viscosity were further explored using the CCD of RSM. The design matrix and the corresponding experimental data to determine the effects of three independent variables are shown in Table 3. By applying multiple regression analysis to the experimental data (Table 5), the following second-order polynomial equation was established:
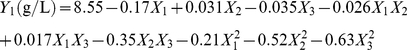
(5)

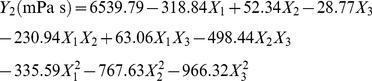
(6)where *Y*_1_ was the EPS production, *Y*_2_ the broth viscosity, *X*_1_ the lactose, *X*_2_ the peptone, and *X*_3_ the temperature. The fit of the model was checked by the coefficient of determination *R* ^2^, which was 0.9758 for EPS production and 0.9674 for broth viscosity, indicating that 97.58% and 96.74% of the variability in the response could be explained by the model. The statistical significance of the second-order model equation was determined with the *F*-test analysis of variance. The “Model *F*-value” of 44.78 (EPS production) and 32.96 (broth viscosity) indicated that the models were significant, and there was only a 0.01% chance that a “Model *F*-Value” this large could occur due to noise (*p*<0.0001). Moreover, the “Lack of Fit *F*-value” was 3.09 (EPS production) and 3.07 (broth viscosity) and the “Lack of Fit *p*-value” was 0.1207 (EPS production) and 0.1221 (broth viscosity), respectively, indicating that the Lack of Fit were not significant relative to the pure error. The coefficient of variation (CV) is the ratio of the standard error of estimate to the mean value of the observed response, and as a general rule a model can be considered reasonably reproducible if the CV is not greater than 10%. Here, the low values of CV (2.17% and 5.59%) indicated great reliabilities of the experiments performed. All these results showed a good agreement between the experimental and predicted values and implied that the mathematical models were suitable for the simulation of EPS production and broth viscosity in the present study.

**Table 5:** Variance analysis of response surface quadratic model for EPS production and broth viscosity of strain SM-A87.

Source	df	EPS production^a^				Broth viscosity^b^			
		Sum of Squares	Mean Square	*F* Value	*p*-value Prob > *F*	Sum of Squares	Mean Square	*F* Value	*p*-value Prob > *F*
Model	9	11.00	1.22	44.78	<0.0001^c^	2.438E+007	2.708E+006	32.96	<0.0001^c^
*X*_1_	1	0.38	0.38	13.86	0.0040^c^	1.388E+006	1.388E+006	16.90	0.0021^c^
*X*_2_	1	0.013	0.013	0.47	0.5096	37419.36	37419.36	0.46	0.5151
*X*_3_	1	0,017	0.017	0.63	0.4459	11306.90	11306.90	0.14	0.7184
*X*_1_ *X*_2_	1	0.55	0.55	20.17	0.0012^c^	4.267E+005	4.267E+005	5.19	0.0459^c^
*X*_1_ *X*_3_	1	0.24	0.24	8.93	0.0136^c^	31815.03	31815.03	0.39	0.5477
*X*_2_ *X*_3_	1	0.97	0.97	35.37	0.0001^c^	1.988E+006	1.988E+006	24.19	0.0006^c^
*X*_1_^2^	1	0.63	0.63	22.96	0.0007^c^	1.623E+006	1.623E+006	19.75	0.0012^c^
*X*_2_^2^	1	3.83	3.83	140.47	<0.0001^c^	8.492E+006	8.492E+006	103.35	<0.0001^c^
*X*_3_^2^	1	5.68	5.68	207.98	<0.0001^c^	1.346E+007	1.346E+007	163.77	<0.0001^c^
Residual	10	0.27	0.027			8.217E+005	82170.07		
Lack of Fit	5	0.21	0.041	3.09	0.1207	6.196E+005	1.239E+005	3.07	0.1221
Pure Error	5	0.067	0.013			2.021E+005	40413.87		
Cor Total	19	11.27				2.520E+007			

a *R* ^2^ = 0.9758; Adj *R* ^2^ = 0.9540; CV = 2.17%.

b *R* ^2^ = 0.9674; Adj *R* ^2^ = 0.9380; CV = 5.59%.

c Model terms are significant.

**Figure 2:**
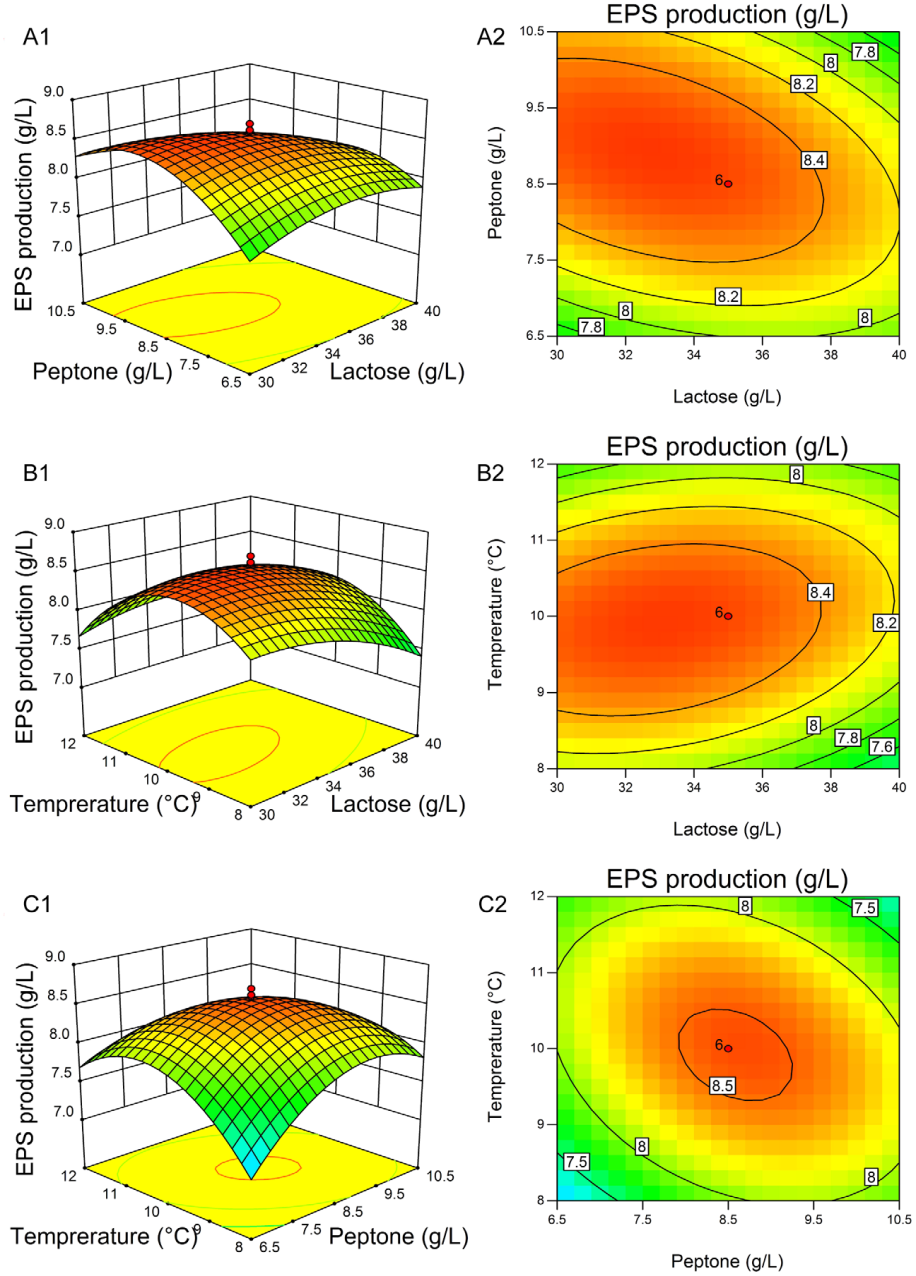
Three-dimensional plots and corresponding contour plots of the effect of three variables on EPS production. When the effect of two variables was plotted, the other variable was set at the central level. A, interaction of lactose and peptone; B, interaction of lactose and temperature; C, interaction of peptone and temperature.

**Figure 3:**
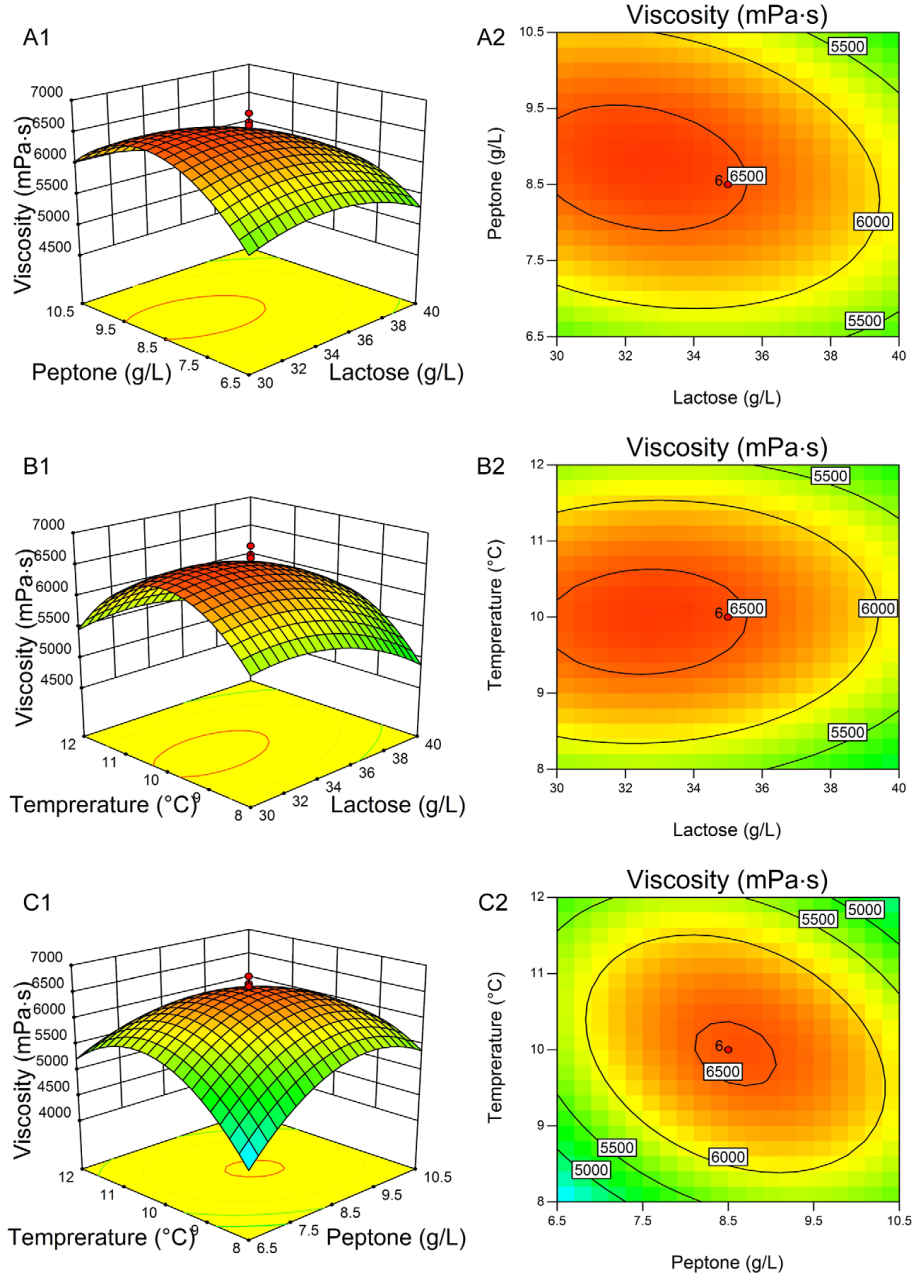
Three-dimensional plots and corresponding contour plots of the effect of three variables on broth viscosity. When the effect of two variables was plotted, the other variable was set at central levels. A, interaction of lactose and peptone; B, interaction of lactose and temperature; C, interaction of peptone and temperature.

The three-dimensional response surfaces and contour plots are shown in Figure 2 (EPS production) and Figure 3 (broth viscosity), which depict the interactions between the two variables by keeping the other variables at their zero levels. The shapes of the contour plots, circular or elliptical, indicate whether the mutual interactions between the variables are significant or not. A circular contour plot of response surfaces indicates that the interaction between the corresponding variables can be ignored, while an elliptical or saddle nature of the contour plot suggests that the interaction between the corresponding variables is significant [21], [22]. In this case, the mutual interactions between every two of the three variables were significant. By solving the inverse matrix using Expert-Design software, the optimal values for both EPS production and broth viscosity of three variables in uncoded units were 32.22 g/L for lactose, 8.87 g/L for peptone and 9.76°C for incubation temperature, respectively. Under the optimum condition, the predicted maximum EPS production was 8.61 g/L and the predicted maximum broth viscosity was 6625.24 mPa•s.

### Verification of optimum conditions

**Figure 4:**
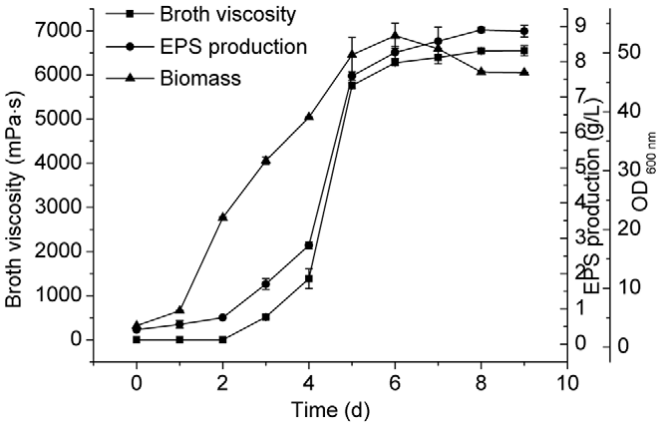
The fermentation process of strain SM-A87 cultured under the predicted optimum culture conditions. The EPS production, broth viscosity and cell growth were detected with time. The culture medium consisted of 32.21 g/L lactose, 8.87 g/L peptone, 1 g/L yeast extract. The pH was adjusted to 8.0, the incubation temperature was 9.8°C, and the culture volume was 100 mL/500 mL. The graph shows data from triplicate experiments (mean ± S.D.).

To validate the adequacy of the model equation, three confirmation experiments were carried out under the optimal medium composition mentioned above. As shown in Figure 4, the cell growth reached the maximum biomass on the 6th day, while the maximum EPS yield at 8.90 g/L and the highest broth viscosity at 6551 mPa•s were attained on the 8th day and the 9th day, respectively. This result indicated that the experimental values agreed with the predicted values well. By optimization of the medium composition and the culture conditions using RSM, the production of EPS was enhanced from 6.47 g/L to 8.90 g/L, and broth viscosity from 3575 mPa•s to 6551 mPa•s. To our knowledge, this is the first report of such high yield of EPS from a marine bacterium [3], [23]–[30].

### EPS properties

**Figure 5:**
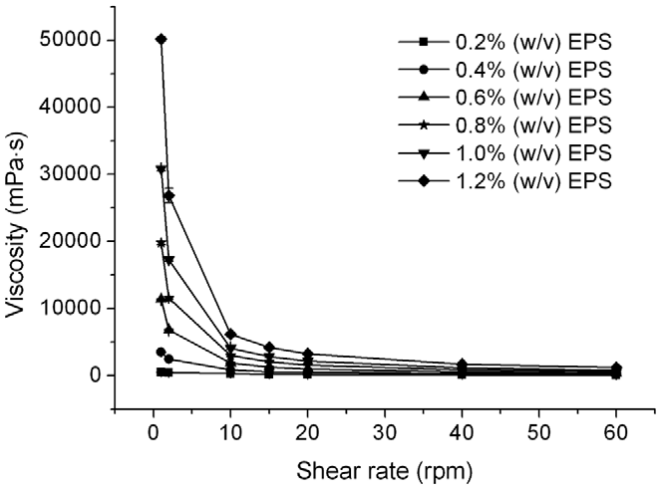
Viscosity of different concentration of crude EPS solution under different shear rates. Viscosity was measured by Brookfield viscometer with the small sample adapter and spindle S16 at 25°C. The graph shows data from triplicate experiments (mean ± S.D.).

Flow curves of the crude EPS aqueous solution at different concentrations with increasing shear rate are shown in Figure 5. The viscosity of the EPS went up with concentration at the same shear rate, which showed its typical non-Newtonian behavior in solution. Within the available shearing rate range, the aqueous dispersions of the EPS exhibited a strong shearing-thinning behavior with pseudoplastic properties. The viscosity of all solutions went down with the shear rate increasing, but the effect of shear rate on viscosity was markedly decreased in diluted solutions.

**Figure 6:**
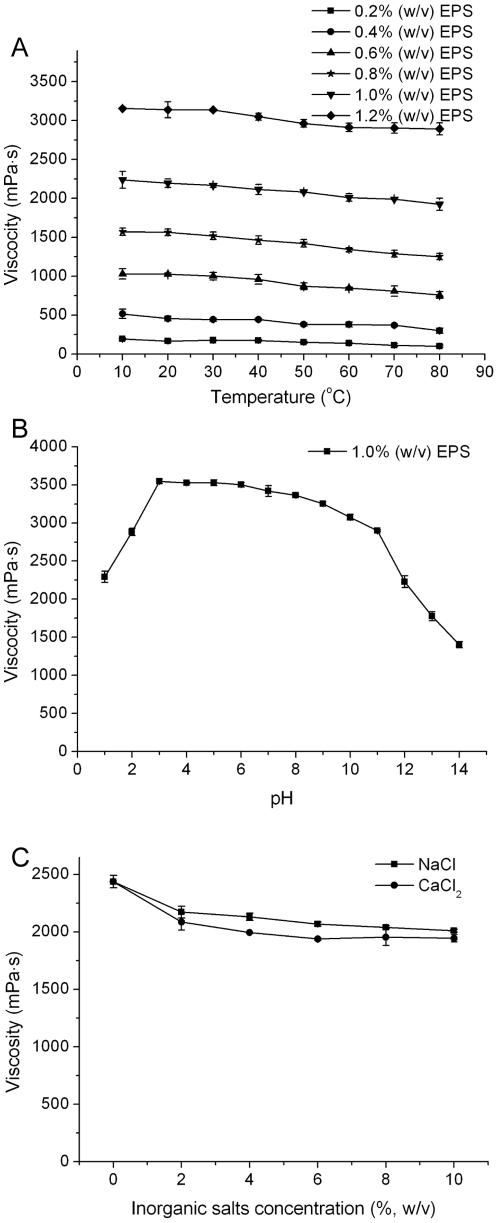
Effect of temperature, pH and inorganic salts on the viscosity of crude EPS solution. Viscosity was measured by a Brookfield viscometer with the small sample adapter and the spindle S16. A, the effect of temperature on the EPS solution of different concentrations (0.2%, 0.4%, 0.6%, 0.8%, 1.0%, and 1.2% (w/v)); B, the effect of different pH (1–14) on 1% (w/v) EPS solution (25°C); C, the effect of different concentration of NaCl and CaCl_2_ (0–10%) on 1% (w/v) EPS solution (25°C). The graph shows data from triplicate experiments (mean ± S.D.).

The EPS from strain SM-A87 had good stability under the conditions of high temperature, wide range of pH and high concentration of salts (Figure 6). When temperature increased from 10°C to 80°C, the apparent viscosity of 1.2% (w/v) EPS solution decreased only about 2% (Figure 6A), indicating that the EPS did not degrade under high temperature and had good thermal stability. The viscosity of the EPS solution had little changes and remained at a high viscosity in the range of pH 3–11, but decreases sharply at pH<3 or pH>11 (Figure 6B). According to the literature reports, the initial increase of the viscosity with pH might be due to the increase of the ionization degree of carboxylate groups in polysaccharide molecules [31], [32], and the subsequent decrease of the viscosity was attributed to the screening effect of excess alkali on the negatively charged polysaccharide molecules [33]. When 2% (w/v) salts was added, the viscosity of the EPS solution decreased slightly, and it almost changed no longer when the salts concentration further increased. The apparent viscosity remained 83% when NaCl concentration increased to 10% (w/v), and about 80% when 10% CaCl_2_ (w/v) was added (Figure 6C). These results showed that the EPS from strain SM-A87 could maintain its stability in the high concentration of salts, indicating that the EPS solution is of good salt resistance.

## Discussion

Carbon source is one of the most important factors affecting EPSs production [34]. A wide variety of carbon sources, including sucrose, glucose, lactose, maltose, mannitol, sorbitol, whey, starch, and even nonsugar sources like methanol and C9 to C16 n-alkanes, can be used to produce microbial EPSs [5]. Many studies demonstrate the influence of the type of carbon source on EPSs production [35]–[37]. And the carbon source that leads to high cell weight does not always result in EPS production increase [5]. In this study, lactose was the best carbon source for EPS production of strain SM-A87. Previous study showed that strain SM-A87 genome harbors 5 predicted β-galactosidase coding genes [13], which suggests that it may efficiently use lactose as a carbon source for EPS synthesis.

It is reported that EPS production is favored by a high carbon : nitrogen ratio [35], and 10∶1 is considered to be the most favorable for EPS production [5]. In this study, it was found that when the concentration of lactose, peptone and yeast extract were 32.22 g/L, 8.87 g/L and 5 g/L, respectively, and the carbon : nitrogen ratio was about 12∶1, the EPS production and broth viscosity were reached maximum. Incubation temperature lower than the optimum may cause enhancement of EPS production and reduction of growth rate and cell mass, resulting in long logarithmic phase of growth and higher viscosity [38]. For instance, the production of EPS from *Pseudoalteromonas* sp. strain CAM025, a marine bacterium isolated from the sea ice of the Southern Ocean, increased approximately 30 fold at −2°C and 10°C than at 20°C [39]. In the current study, a similar result was obtained. The production of EPS from strain SM-A87 reached its maximum at 9.8°C after 8 d. It was 6.5 fold of the yield at 35°C, the optimum growth temperature of strain SM-A87 (data not shown). Sutherland proposed a mechanism to explain this phenomenon, *i.e.* a decrease in temperature causes a decrease in growth rate and cell wall polymer biosynthesis, resulting in more precursors available for EPSs synthesis [40].

With the exception of hydrothermal vents, most deep-sea areas are regarded as extreme environments with low nutrient concentration, low temperature and high pressure [25]. Deep-sea bacteria depend for their sustenance on the rain of particulate organic matter produced by photosynthesis in the photic layer, although only about 1–3% of photosynthetically produced organic matter reaches the deep-sea floor [41]. In the organic matter in deep-sea sediment, organic nitrogen (protein) is generally present in extremely low concentrations, thus representing a limiting factor to the distribution, metabolism and growth of deep-sea bacteria [42]. And disappearance of nitrogen from the medium is a signal for EPS synthesis, as observed for the synthesis of pullulan and scleroglucan [34]. As organic nitrogen concentration is extremely low in deep-sea sediment, high carbon : nitrogen ratio is an obvious nutrition characteristic for deep-sea bacteria . It was reported that the carbon : nitrogen ratios of the surficial deep-sea sediments at three sites with different depths in the North-East Atlantic were in the ranges 6.7–9.0 [43]. Low temperature is another environment condition in deep-sea sediment. Except for hydrothermal vents, most deep-sea environments maintain at 3°C (±1°C) [44]. These environmental conditions are consistent with the favorable conditions for the EPS production of strain SM-A87, suggesting that this strain may secrete large quantity of EPS *in situ*.

In the marine environment, EPSs play important roles in the production of aggregates, adhesion to and colonization of surfaces, biofilm formation, sequestering of nutrients, and thus provide protection and ecosystem stability [3]. Comparison of some deep-sea bacteria with sequenced genomes revealed that they all have EPS biosynthesis genes, suggesting that EPS production may be a common strategy that deep-sea bacteria adopt to endure extreme conditions [45]. By now, it is still unclear how the EPS from strain SM-A87 may act as organic ligands, protectants against low temperature, and how the structure of the EPS is related to its ecological role. Further work is necessary to define the structure of the EPS and to study the relationship of the structure and the function of the EPS.

Pseudoplastic or shear thinning behavior has been reported for other biopolymers with industrial applications, such as xanthan [46] and gellan [47]. Shear thinning behavior of a polysaccharide has several potential advantages in food applications and pseudoplasticity is important in helping to provide good sensory qualities, such as flavor release, mouth feel and suspension properties of food products [48]. In addition, a reduction in viscosity with shear rate increasing becomes favorable in industrial operations such as mixing and pumping [49]. The high viscosity, strong shearing-thinning behavior with pseudoplastic properties, good thermal and pH stability and salt resistance of the SM-A87 EPS solution suggest that the EPS from strain SM-A87 may have good potential for biotechnological applications.
